# The diagnostic value of non-invasive methods for diagnosing bladder outlet obstruction in men with lower urinary tract symptoms: A meta-analysis

**DOI:** 10.3389/fsurg.2022.986679

**Published:** 2022-09-20

**Authors:** Yu Cheng, Taicheng Li, Xiaoyu Wu, Qin Ling, Ke Rao, Xiaoyi Yuan, Zhong Chen, Guanghui Du, Shengfei Xu

**Affiliations:** Department of Urology, Tongji Hospital, Tongji Medical College, Huazhong University of Science and Technology, Wuhan, China

**Keywords:** lower urinary tract symptoms, bladder outlet obstruction, non-invasive methods, diagnosis, meta-analysis

## Abstract

**Purpose:**

We conducted the first meta-analysis to determine the diagnostic value of non-invasive methods for diagnosing bladder outlet obstruction (BOO) in men with lower urinary tract symptoms (LUTS).

**Methods:**

We searched a range of databases for relevant publications up to June 2022, including PubMed, Embase, Web of Science, and the Cochrane Library. Retrieved studies were then reviewed for eligibility and data were extracted. The risk of bias (RoB) was assessed using the QUADAS-2 tool. We then performed a formal meta-analysis to evaluate the accuracy of various non-invasive methods for diagnosing BOO in men.

**Results:**

We identified 51 eligible studies including 7,897 patients for meta-analysis. The majority of the studies had a low overall RoB. Detrusor wall thickness (DWT) (pooled sensitivity (SSY): 71%; specificity (SPY): 88%; diagnostic odds ratio (DOR): 17.15; area under curve (AUC) 0.87) and the penile cuff test (PCT) (pooled SSY: 87%; SPY: 78%; DOR: 23.54; AUC: 0.88) showed high accuracy for diagnosing BOO. Furthermore, data suggested that DWT had the highest pooled SPY (0.89), DOR (32.58), and AUC (0.90), when using 2 mm as the cut-off.

**Conclusion:**

Of the non-invasive tests tested, DWT and PCT had the highest levels of diagnostic accuracy for diagnosing BOO in men with LUTS. DWT, with a 2 mm cut-off, had the highest level of accuracy. These two methods represent good options as non-invasive tools for evaluating BOO in males.

## Introduction

Lower urinary tract symptoms (LUTS) can be very troublesome for both male and female patients and can cause a reduction in their quality of life. Most patients seek medical help due to bothersome LUTS, especially when they develop bladder outlet obstruction (BOO) as this can result in severe urinary difficulty (1). BOO is mainly caused by benign prostatic hyperplasia (BPH) and usually requires surgical treatment. Therefore, it is very important that we can determine whether LUTS is due to BOO if we are to optimize patient management (2). Over recent years, the evaluation of LUTS/BPH has depended heavily on pressure-flow study (PFS) of urodynamic study (UDS) as the gold standard diagnostic tests for BOO (3). However, UDS has several disadvantages. First, PFS requires transurethral intubation and may cause urinary symptoms, such as hematuria and urinary tract infections. Furthermore, PFS can be unpleasant and is commonly associated with anxiety and embarrassment (4). In addition, UDS is expensive, time consuming, and requires delicate instruments and specific expertise.

Given the invasive nature and side effects associated with conventional invasive PFS, a variety of non-invasive diagnostic methods have been developed (5). Although these novel and non-invasive diagnostic methods were designed to improve the quality of life in patients with LUTS by promoting earlier diagnosis and treatment, and do show significant potential (6), there is some conflict with regards to their specific clinical outcomes. Previous authors have evaluated and summarized the diagnostic value of these non-invasive methods (5, 7–14); nevertheless, researchers have yet to perform a meta-analysis to investigate the diagnostic accuracy for these approaches in a quantitative manner.

The aim of the present meta-analysis was to re-evaluate and determine the diagnostic accuracy of non-invasive methods for the diagnosis of BOO in men with LUTS by assessing sensitivity (SSY), specificity (SPY), diagnostic odds ratio (DOR) and area under curve (AUC). This was the first meta-analysis to quantitatively compare the diagnostic value of different non-invasive methods for BOO.

## Materials and methods

This meta-analysis was conducted based on Preferred Reporting Items for a Systematic Review and Meta-Analysis (PRISMA). Neither ethical approval or informed consent was required for this study.

### Search strategy

We searched a range of databases for relevant publications up to June 2022, including PubMed, Embase, Web of Science, and the Cochrane Library. The search strategy was (“uroflowmetry” OR “flow rate” OR “intravesical prostat* protrusion” OR “intravesical protrusion” OR “penile cuff” OR “urocuff” OR “detrusor wall thickness” OR “detrusor thickness” OR “bladder wall thickness” OR “bladder thickness” OR “external condom catheter” OR “doppler ultrasound” OR “resistive index” OR “velocity ratio” OR “bladder weight” OR “prostate volume” OR “international prostat* symptom* score” OR “IPSS” OR “residual urine” OR “post-void* residual urine” OR “RUV” OR “PVR” OR “prostat* specific antigen” OR “PSA” OR “near-infrared spectroscopy” OR “noninvasive” OR “non-invasive” OR “noninvasively” OR “non-invasively”) AND (“bladder obstruction” OR “benign prostatic obstruction” OR “bladder outlet obstruction” OR “bladder outflow obstruction” OR “BOO” OR “BPO” OR “infravesical obstruction”). To achieve a comprehensive literature search, we also reviewed the reference lists of the retrieved literature. The articles included in this study were restricted to human subjects and those published in English. Two researchers carried out the same literature screening protocols; any disagreements were resolved by a third researcher.

### Eligibility criteria

Following the removal of duplicate articles, two researchers independently reviewed the titles and abstracts of the retrieved studies. To be eligible for analysis, the articles needed to meet our specific inclusion criteria: (1) population: patients with LUTS aged ≥18 years; (2) index test: non-invasive methods. The following noninvasive tests were eligible for inclusion in meta-analysis: the penile cuff test (PCT), near-infrared spectrum (NIRS), ultrasonography of post-voided residual (PVR), intravesical prostatic protrusion (IPP), detrusor wall thickness (DWT), bladder wall thickness (BWT), resistive index (RI), prostate volume (PV), and free uroflowmetry, a detailed description of each index test is included in the Supplementary Material; (3) reference standard: invasive PFS; (4) outcome: diagnostic accuracy for the diagnosis of BOO; (5) study design: any type, including comparative studies, clinical trials, retrospective or prospective studies; (6) complete data: all data could be obtained directly or calculated and included true positive (TP), false positive (FP), true negative (TN), and false negative (FN) data. The exclusion criteria were as follows: (1) failure to meet the inclusion criteria; (2) duplicated publications; (3) reviews, case reports, conference abstracts, letters and editorials; (4) non-English and non-human studies.

### Data extraction

All data extraction was completed by two researchers independently and manually according to the inclusion criteria, any inconsistency was resolved by a third researcher. We extracted a range of data from eligible publications, including: (a) author-year; (b) study design; (c) mean age; (d) country; (e) sample size; (f) index test; (g) cut-off value; (h) TP; (i) FP; (j) TN; and (k) FN.

### Quality assessment

The quality of all studies included in this meta-analysis was assessed by two researchers in accordance with the Quality Assessment of Diagnostic Accuracy Studies 2 (QUADAS-2) tool (15). This tool was used to evaluate the risk of bias (RoB) on the basis of the following criteria: patient selection, index test, reference standard, flow and timing, and assessing applicability concerns by patient selection, index test, reference test.

### Statistical analysis

To determine the diagnostic accuracy of non-invasive tests for the diagnosis of BOO, we adopted pooled SSY, SPY, DOR and AUC of summary receiver operating characteristics (SROC) as the primary indicators. Because these four indicators can well illustrate the diagnostic ability of the index tests (16–18). The SSY represents the ability to detect disease and the SPY represents the ability to exclude a disease. The DOR is a measure for the discriminative power of a diagnostic test: the ratio of the odds of a positive test result among diseased to the odds of a positive test result among the non-diseased. SROC curves are used to determine test performances and present the tradeoff between the SSY and SPY of non-invasive tests. A two-by-two contingency table (consisting of TP, FP, FN and TN) was then constructed based on the data extracted from each study included in the meta-analysis. If a study used different cut-off values for the same index test, we adopted data for the most common cut-off value to conduct the meta-analysis. A bivariate model was then used to calculate the pooled SSY, SPY, DOR and AUC, along with 95% confidence intervals (CIs) (19). Pooled data was displayed using forest plots and summary receiver operating characteristics (SROC) plots. The heterogeneity of the pooled data was assessed by Cochrane's *Q* test and *I*^2^ test (20). If the data showed little heterogeneity (*P* ≥ 0.1 and *I*^2^ < 50%), a fixed-effect model was used; otherwise, a random-effect model was adopted. A threshold effect was determined by calculating Spearman's correlation coefficient between SSY and the false positive rate (1-SPY) (18); a strong positive correlation was considered a significant threshold effect. Sensitivity analysis and meta-regression were also conducted to explore the sources of heterogeneity relating to non-threshold effects. Publication bias was evaluated with Deeks’ funnel plots and an associated regression test of asymmetry (21). Data were analyzed by STATA version 14.0 (StataCorp LP, College Station, TX, USA) using midas commands. Spearman's correlation coefficient was calculated by MetaDiSc 1.4 (Universidad Complutense, Madrid, Spain). Quality assessment was performed by RevMan 5.3 (Cochrane Collaboration, Oxford, UK). *P *< 0.05 was considered statistically significant.

## Results

### Literature searches and study characteristics

**Figure 1:**
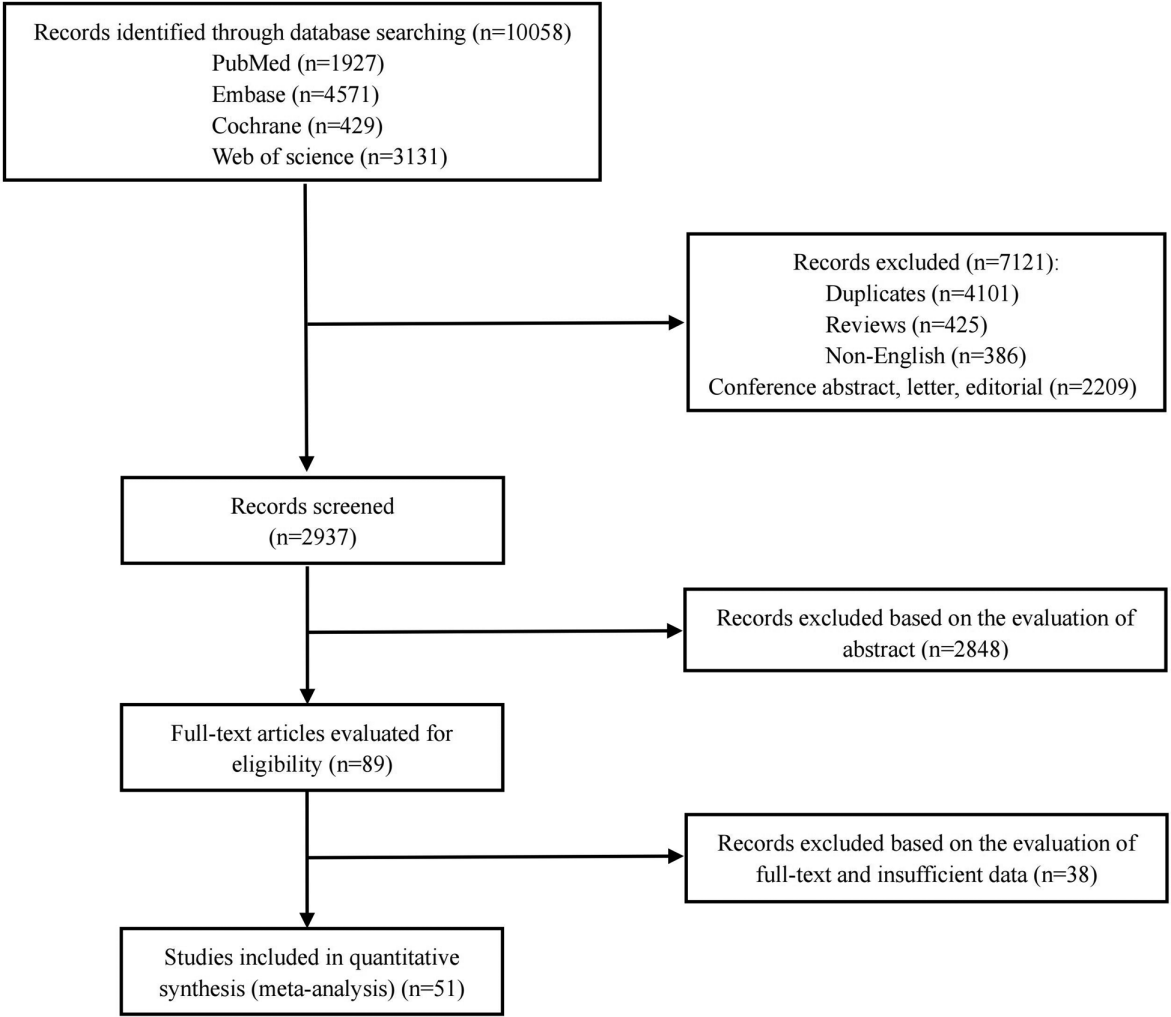
The screening flow diagram of the retrieval studies.

We conducted a comprehensive literature search of the PubMed, Embase, Web of Science and the Cochrane Library databases following an established search strategy. We identified a total of 10,058 articles. After the removal of duplicates, reviews, case reports, conference abstracts, letters, and editorials, 2,937 articles were left for screening. Then, 2,886 studies were removed following abstract and full-text evaluation and by insufficient data. For BWT, one study resulted in a significant increase in heterogeneity, after excluding this study, the final number of studies was not enough to perform meta-analysis. For NIRS, the method and calculation in each of the study was different from the other and far from being standard, Therefore, we did not include these two index tests. Finally, 51 articles were eligible for meta-analysis (22–72). Full details of the screening process are shown in Figure 1.

Of the 51 studies included in this meta-analysis, the publication year ranged from 1994 to 2021. These studies involved 21 countries and a total of 7,897 participants. All of the 51 studies were non-randomized trials (NRT); 22 studies were prospective, two were retrospective; 14 studies were blinded, 13 were not blinded; and the remaining studies did not describe their specific design. All 51 studies examined the accuracy of non-invasive methods for the diagnosis of BOO in men with LUTS, using PFS as the gold standard. For specific descriptions of the included studies, see Table 1.

**Table 1:** Characteristics of the included studies.

Author-year	Country	Study design	Patients number	Age (mean)	Index test	Cut-off	TP	FP	FN	TN
Poulsen 1994 (22)	Denmark	NR, NRT, No	153	68	Qmax	10 ml/s	68	23	31	31
			153		Qmax	15 ml/s	89	37	10	17
Comiter 1996 (23)	American	NR, NRT, NR	205	68.3	Qmax	12 ml/s	80	27	23	75
Reynard 1996 (24)	England	NR, NRT, No	165	NR	Qmax	8 ml/s	43	10	57	56
			165		Qmax	10 ml/s	71	19	29	46
			165		Qmax	12 ml/s	83	33	16	33
			165		Qmax	15 ml/s	95	42	5	23
Ding 1997 (25)	Singapore	PS, NRT, NR	126	75	PVR	50 ml	17	30	31	48
DuBeau 1998 (26)	American	NR, NRT, No	99	72.4	PVR	50 ml	60	19	7	13
			99		PVR	100 ml	53	18	14	14
			99		PVR	200 ml	47	14	20	18
			99		Qmax	10 ml	37	9	30	23
Reynard 1998 (27)	England	NR, NRT, No	897	66.5	Qmax	10 ml/s	252	107	288	250
			897		Qmax	15 ml/s	440	221	100	136
Kuo 1999 (28)	Tai wan	PS, NRT, No	324	67.2	PV	20 ml	104	33	108	79
			324		PV	40 ml	35	2	177	110
			324		PVR	50 ml	37	9	175	103
			324		PVR	100 ml	31	5	181	107
			324		Qmax	10 ml/s	135	44	77	68
			324		Qmax	15 ml/s	179	75	33	37
Rasmussen 1999 (29)	Denmark	NR, NRT, NR	29	66	PVR	50 ml	0	2	15	12
			29		Qmax	10 ml/s	5	0	10	14
			29		Qmax	15 ml/s	9	8	6	6
Kojima 2000 (30)	Japan	NR, NRT, NR	57	NR	RI	0.7	28	13	5	11
Steele 2000 (31)	American	PS, NRT, NR	204	66.7	PV	40 ml	100	19	52	33
			204		Qmax	10 ml/s	111	21	41	31
Sullivan 2000 (32)	American	NR, NRT, No	90	NR	PCT	PCR index 100%	39	17	4	40
Oelke 2002 (33)	Germany	NR, NRT, NR	70	63	DWT	2 mm	21	1	12	36
			70		PV	20 ml	30	27	3	10
			70		PVR	50 ml	27	21	6	16
			70		Qmax	15 ml/s	33	27	0	10
Watanabe 2002 (34)	Japan	PS, NRT, No	51	66.4	PV	30 ml + H:W 0.8	10	0	14	27
Blenky 2003 (35)	Israel	PS, NRT, Yes	29	65.6	RI	0.7	19	1	3	6
Chia 2003 (36)	Singapore	PS, NRT, Yes	200	64.6	IPP	10 mm	95	6	30	69
			200		IPP	5 mm	116	41	9	34
			200		PV	30 ml	99	34	26	41
			200		PVR	100 ml	93	7	32	68
			200		Qmax	10 ml/s	113	39	12	36
Salinas 2003 (37)	Spain	NR, NRT, Yes	52	54.1	PCT	nomogram	34	8	0	10
Aganovic 2004 (38)	B/H	NR, NRT, NR	102	64.68	Qmax	10 ml/s	47	3	30	22
Harding 2004 (39)	England	NR, NRT, Yes	101	63	PCT	PCR index 160%	25	11	7	58
			101		Qmax	10 ml/s	26	25	6	44
Griffiths 2005 (40)	England	NR, NRT, No	144	NR	PCT	Griffiths nomogram	36	17	20	71
Nose 2005 (41)	Japan	NR, NRT, Yes	30	62.5	IPP	10 mm	9	8	1	12
Kessler 2006 (42)	Switzerland	NR, NRT, No	102	67	DWT	1.5 mm	61	35	0	6
			102		DWT	2 mm	56	13	5	28
			102		DWT	2.5 mm	43	5	19	36
			102		DWT	2.9 mm	26	0	35	41
Lim 2006 (43)	Singapore	PS, NRT, NR	95	66	IPP	5 mm	40	25	7	23
			95		IPP	10 mm	22	9	25	39
			95		PV	20 ml	43	36	4	12
			95		PV	40 ml	24	12	23	36
Oelke 2007 (44)	Netherland	PS, NRT, Yes	160	62	DWT	2 mm	68	4	13	81
			160		PV	25 ml	64	62	11	23
			160		PVR	50 ml	54	49	21	36
			160		Qmax	10 ml/s	51	23	24	62
			160		Qmax	15 ml/s	74	52	1	33
Reis 2008 (45)	Brazil	PS, NRT, Yes	42	64.8	IPP	5 mm	19	11	1	11
			42		IPP	10 mm	16	7	4	15
Ku 2009 (46)	Korea	NR, NRT, No	212	67.5	PV	35 ml	47	66	10	89
			212		PV	40 ml	42	54	15	101
			212		PV	45 ml	37	41	20	114
			212		Qmax	10 ml/s	33	53	24	102
			212		Qmax	12 ml/s	44	71	13	84
			212		Qmax	15 ml/s	54	112	3	43
Franco 2010 (47)	Italy	PS, NRT, Yes	100	67	DWT	6 mm	54	5	20	21
			100		IPP	12 mm	48	6	26	20
			100		PV	38 ml	53	10	21	16
Abdel-Aal 2011 (48)	Egypt	NR, NRT, Yes	85	58.7	DWT	2 mm	23	12	12	38
			85		IPP	8 mm	28	10	7	40
			85		PV	45 ml	30	37	5	13
Pascual 2011 (49)	Spain	PS, NRT, No	39	63.1	IPP	10.5 mm	19	5	2	13
Aganovic (a) 2012 (50)	B/H	NR, NRT, NR	111	65.4	IPP	10 mm	32	11	22	46
Aganovic (b) 2012 (51)	B/H	PS, NRT, NR	110	65.3	IPP	12 mm	37	9	25	39
Aldaqadossi 2012 (52)	Egypt	PS, NRT, Yes	338	65	RI	0.71	134	39	24	141
Hossain 2012 (53)	Bangladesh	NR, NRT, NR	50	64.3	IPP	10 mm	18	5	8	19
			50		PV	40 ml	15	8	11	16
Zhang 2012 (54)	China	PS, NRT, Yes	74	69.9	RI	0.69	40	3	11	20
Elsaied 2013 (55)	Egypt	NR, NRT, Yes	50	61.7	DWT	2 mm	19	2	4	25
			50		PV	25 ml	20	19	3	8
			50		PVR	50 ml	17	15	6	12
			50		Qmax	10 ml/s	23	17	0	10
Shin 2013 (56)	Korea	RS, NRT, NR	239	69.9	IPP	5.5 mm	31	38	15	155
			239		PV	30 ml	30	80	16	113
			239		PVR	50 ml	23	20	23	173
			239		Qmax	10 ml/s	37	100	9	93
Bianchi 2014 (57)	Italy	NR, NRT, No	48	61.5	PCT	Griffiths nomogram	21	10	0	17
Zhang 2014 (58)	China	PS, NRT, Yes	55	65.7	PV	54.4 ml	41	7	2	5
Kazemeyni 2015 (59)	Iran	NR, NRT, NR	51	65.5	PCT	Griffiths nomogram	16	8	2	25
Matulewicz 2015 (60)	American	NR, NRT, No	19	NR	PCT	Modified ICS nomogram	12	1	4	2
Ahmed 2016 (61)	Arabia	PS, NRT, NR	157	65	IPP	10.9 mm	87	6	22	42
Lee 2016 (62)	Singapore	NR, NRT, NR	61	66	IPP	5 mm	14	27	0	20
			61		IPP	10 mm	8	11	6	36
Suzuki 2016 (63)	Japan	RS, NRT, NR	350	68.9	IPP	10 mm	135	42	45	128
			350		PV	40.1 ml	109	50	71	120
			350		RI	0.726	124	68	56	102
Farag 2017 (64)	Egypt	NR, NRT, NR	72	63.0	Qmax	7 ml/s	31	1	24	16
Ko 2017 (65)	Korea	PS, NRT, Yes	107	67	PCT	Griffiths nomogram	26	22	3	56
Aganovic 2019 (66)	B/H	PS, NRT, NR	135	66.1	PCT	PCR index 96.4%	52	4	18	61
Garg 2019 (67)	India	PS, NRT, NR	240	57.1	DWT	5.5 mm	80	4	88	68
			240		IPP	7.5 mm	146	12	22	60
			240		RI	0.62	132	12	36	60
Reddy 2019 (68)	India	PS, NRT, NR	164	66.72	IPP	5 mm	83	40	8	33
			164		IPP	10 mm	54	11	37	62
Kim 2020 (69)	Korea	NR, NRT, NR	59	69.6	PCT	Modified ICS nomogram	36	0	9	14
Mosawi, 2020 (70)	NR	NR, NRT, NR	63	NR	IPP	10 mm	31	15	7	10
			63		PV	40 ml	21	15	17	10
Park, 2020 (71)	Korea	PS, NRT, NR	196	69.5	DWT	3 mm	36	29	48	83
Wadie, 2021 (72)	Egypt	PS, NRT, NR	459	54	PV	40 ml	105	72	75	193
			459		Qmax	10 ml/s	150	134	135	40
			459		Qmax	15 ml/s	39	66	246	108

TP, true positive; FP, false positive; FN, false negative; TN, true negative; NRT, non-randomized trial; PS, prospective study; RS, retrospective study; No, not blinded; Yes, blinded; NR, not referred; Qmax, maximum flow rate; PVR, post-voided residual; PV, prostate volume; RI, resistive index; PCT, penile cuff test; IPP, intravesical prostatic protrusion; DWT, detrusor wall thickness; B/H, Bosnia and Herzegovina.

### Quality assessment

**Figure 2:**
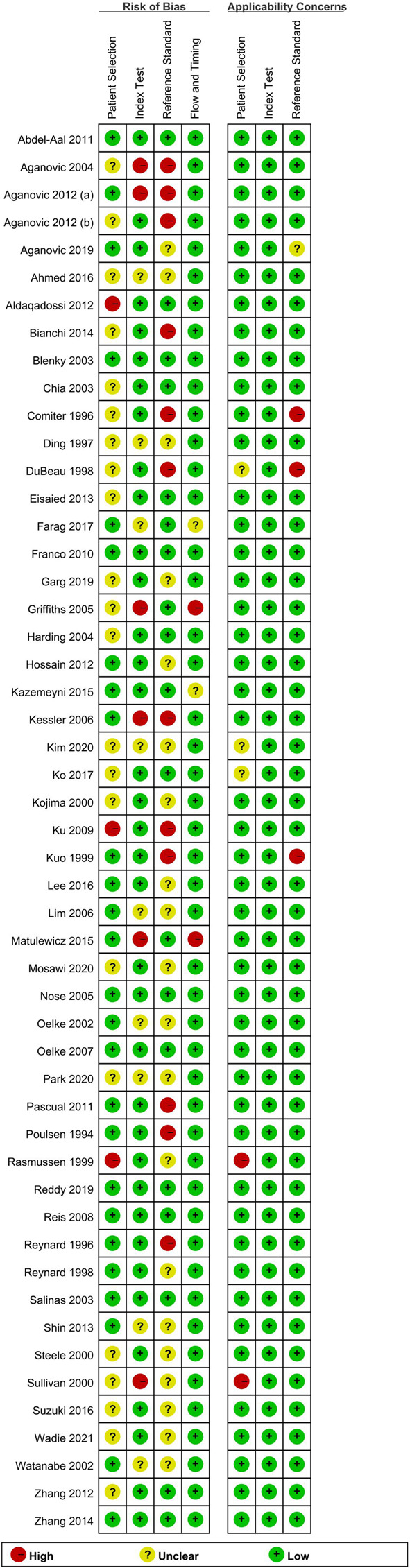
Risks of bias of the included studies based on QUADAS-2 tool.

Outcomes relating to quality assessment and RoB are shown in Figure 2. Overall, the RoB was generally low for most studies. In the patient selection domain, three studies were classified as high risk due to inappropriate inclusion or exclusion criteria. Twenty-three studies had an unclear risk because it was unknown as to whether they adopted consecutive patients or random samples. In the index test domain, 6 studies showed high risk because the results of the index test were interpreted with knowledge of the reference standard. Nine studies were associated with an unclear risk. In the reference standard domain, 12 studies had a high risk due to knowledge of the results of index tests when interpreting the results of the reference standard; the other 20 studies were at an unclear risk. In the flow and timing domain, only two studies had a high risk; this was due to an inappropriate interval between the index test and the reference standard. In terms of applicability concern, the majority of the publications were at low risk, thus indicating that the patients, index test, and reference standard, for most studies were representative of clinical routine practice.

### Quantitative analysis of the results (meta-analysis)

With regards to the index tests (DWT, PCT, RI, IPP, Qmax, PV, PVR), we conducted quantitative analysis (meta-analysis) for these tests. The data extracted or calculated from published studies are presented in Table 1.

#### Diagnostic accuracy results

**Figure 3:**
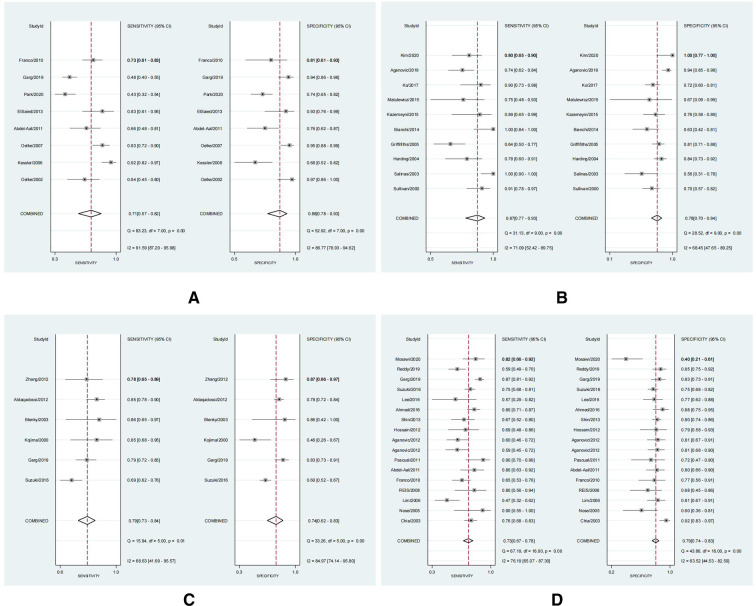
The forest plots of the sensitivity (SSY) and specificity (SPY) with a 95% confidence interval for DWT (**A**), PCT (**B**), RI (**C**), IPP (**D**) in the diagnosis of BOO in men.

**Figure 4:**
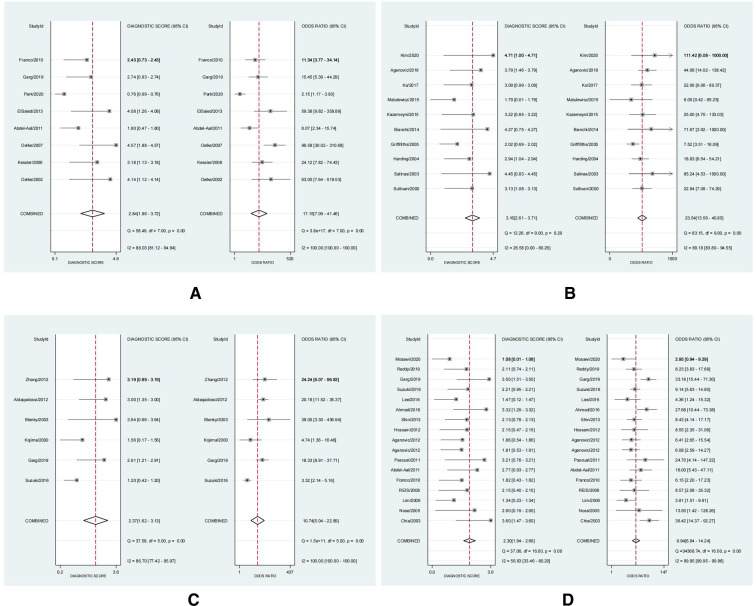
The forest plots of diagnostic odds ratio (DOR) with a 95% confidence interval for DWT (**A**), PCT (**B**), RI (**C**), IPP (**D**) in the diagnosis of BOO in men.

**Figure 5:**
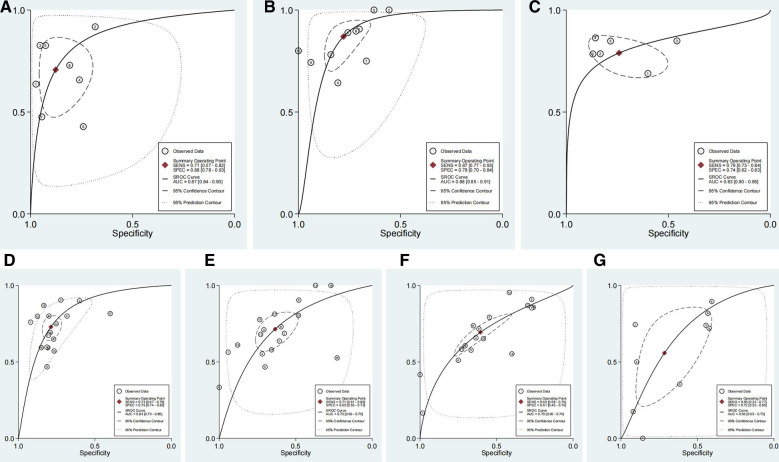
The summary of receiver operator characteristic (SROC) with a 95% confidence interval for DWT (**A**), PCT (**B**), RI (**C**), IPP (**D**), Qmax (**E**), PV (**F**) and PVR (**G**) in the diagnosis of BOO in men.

Outcomes for pooled SSY, SPY, DOR, and AUC of SROC curve are shown in Figures 3–5. Figure 3 shows the pooled SSY and SPY. Across these tests, the pooled SSY ranged from 71% to 87% and the pooled SPY ranged from 74% to 88%. PCT had the highest pooled SSY at 87% (95% CI: 77%–93%) (Figure 3B), followed by RI at 79% (95% CI: 73%–84%) (Figure 3C). DWT had the highest pooled SPY at 88% (95% CI: 78%–93%) (Figure 3A), followed by IPP at 79% (95% CI: 74%–83%) (Figure 3D). The pooled DOR, as presented by forest plots, are shown in Figure 4, ranging from 9.94 to 23.54. PCT exhibited the optimal DOR (23.54, 95% CI: 13.56–40.85) (Figure 4B), followed by DWT at 17.15 (95% CI: 7.09–41.46) (Figure 4A). Due to a significant threshold effect was found for PCT, Qmax and PV, thus we only fit SROC curve and calculate the area under ROC curve for these three tests. SROC curves indicated that DWT and PCT had a relatively better diagnostic power, with AUCs of 0.87 (95% CI: 0.84–0.90) and 0.88 (95% CI: 0.85–0.91) respectively. These indicators are summarized in Table 2. In brief, DWT and PCT showed high levels of diagnostic accuracy; the pooled SSY and SPY exceeded 70%, DORs were the highest and the pooled AUCs exceeded 0.85.

**Table 2:** Summary of the pooled data for each type of the index test.

Test	*n*	Patients	SSY (95% CI)	SPY (95% CI)	DOR (95% CI)	AUC (95% CI)
DWT	8	1,003	0.71 (0.57, 0.82)	0.88 (0.78, 0.93)	17.15 (7.09, 41.46)	0.87 (0.84, 0.90)
PCT	10	806	0.87 (0.77, 0.93)	0.78 (0.70, 0.84)	23.54 (13.56, 40.85)	0.88 (0.85, 0.91)
RI	6	1,088	0.79 (0.73, 0.84)	0.74 (0.62, 0.83)	10.74 (5.04, 22.89)	0.83 (0.80, 0.86)
IPP	17	2,136	0.73 (0.67, 0.78)	0.79 (0.74, 0.83)	9.94 (6.94, 12.24)	0.83 (0.79, 0.86)
Qmax	19	3,911	-	-	-	0.74 (0.70, 0.78)
PV	17	2,767	-	-	-	0.70 (0.66, 0.74)
PVR	9	1,297	-	-	-	0.69 (0.65, 0.73)

SSY, sensitivity; SPY, specificity; DOR, diagnostic odds ratio; AUC, area under curve; PLR, positive likelihood ratio; NLR, negative likelihood ratio; DS, diagnostic score; DWT, detrusor wall thickness; PCT, penile cuff test; RI, resistive index; IPP, intravesical prostatic protrusion; Qmax, maximum flow rate; PV, prostate volume; PVR, post-voided residual.

#### The diagnostic accuracy of each type of test using the most commonly used cut-off

We selected the most commonly used threshold values for each test to perform further analysis. We found that DWT (using 2 mm as the cut-off) possessed the greatest diagnostic accuracy for diagnosing BOO in men across these index tests, with a pooled SSY of 0.79 (95% CI: 0.68–0.88), SPY of 0.89 (95% CI: 0.75–0.96), DOR of 32.58 (95% CI: 12.04–88.17), and an AUC of 0.90 (95% CI: 0.87–0.92). PCT (using Griffith's nomogram as the diagnostic criteria) had the second-best diagnostic accuracy, with a pooled SSY of 0.89 (95% CI: 0.67–0.97), SPY of 0.73 (95% CI: 0.65–0.81), DOR of 22.98 (95% CI: 6.76–77.76) and an AUC of 0.81 (95% CI: 0.78–0.85). Table 3 shows a summary of results for each type of index test using the most commonly threshold values.

**Table 3:** Summary of the pooled data for each type of index test using the most commonly used threshold values.

Test	*n*	Cut-off	SSY (95% CI)	SPY (95% CI)	DOR (95% CI)	AUC (95% CI)
DWT	5	2 mm	0.79 (0.68, 0.88)	0.89 (0.75, 0.96)	32.58 (12.04, 88.17)	0.90 (0.87, 0.92)
PCT	4	Griffiths nomogram	0.89 (0.67, 0.97)	0.73 (0.65, 0.81)	22.98 (6.76, 77.76)	0.81 (0.78, 0.85)
IPP	10	10 mm	0.70 (0.62, 0.77)	0.77 (0.68, 0.84)	7.67 (4.89, 12.01)	0.79 (0.75, 0.83)
Qmax	15	10 ml/s	0.69 (0.59, 0.77)	0.63 (0.52, 0.72)	3.65 (2.30, 5.79)	0.70 (0.66, 0.74)
PV	7	40 ml	0.54 (0.40, 0.68)	0.76 (0.57, 0.88)	3.74 (2.59, 5.42)	0.68 (0.64, 0.72)
PVR	8	50 ml	0.53 (0.28, 0.77)	0.68 (0.47, 0.84)	2.45 (1.30, 4.64)	0.66 (0.61, 0.70)

SSY, sensitivity; SPY, specificity; DOR, diagnostic odds ratio; AUC, area under curve; PLR, positive likelihood ratio; NLR, negative likelihood ratio; DS, diagnostic score; DWT, detrusor wall thickness; PCT, penile cuff test; IPP, intravesical prostatic protrusion; Qmax, maximum flow rate; PV, prostate volume; PVR, post-voided residual.

### Heterogeneity test

The heterogeneity between studies included threshold effects and non-threshold effects. The threshold effect was assessed by Spearman's correlation coefficient, with values of 0.119 (*P* = 0.779) for DWT, 0.673 (*P* = 0.033) for PCT, 0.029 (*P* = 0.957) for RI, 0.383 (*P* = 0.129) for IPP, 0.527 (*P* = 0.025) for Qmax, 0.813 (*P* = 0.000) for PV, and 0.583 (*P* = 0.099) for PVR. Therefore, a statistically significant threshold effect was found for PCT, Qmax and PV and we only fit SROC curve and calculate the area under ROC curve for these three tests (Figures 5E–G; Table 2). The heterogeneity of the non-threshold effect was assessed by Cochrane's *Q* test and *I*^2^ test. Forest plots showed that the heterogeneity for the pooled SSY, SPY and DOR for all of these index tests were generally high (*P* < 0.1 for Cochrane's *Q* test and *I*^2^ > 50% for *I*^2^ test). Therefore, a random effect model was applied when pooling the data. To investigate the potential sources of heterogeneity, we conducted sensitivity analysis and meta-regression analysis.

### Sensitivity analysis

**Figure 6:**
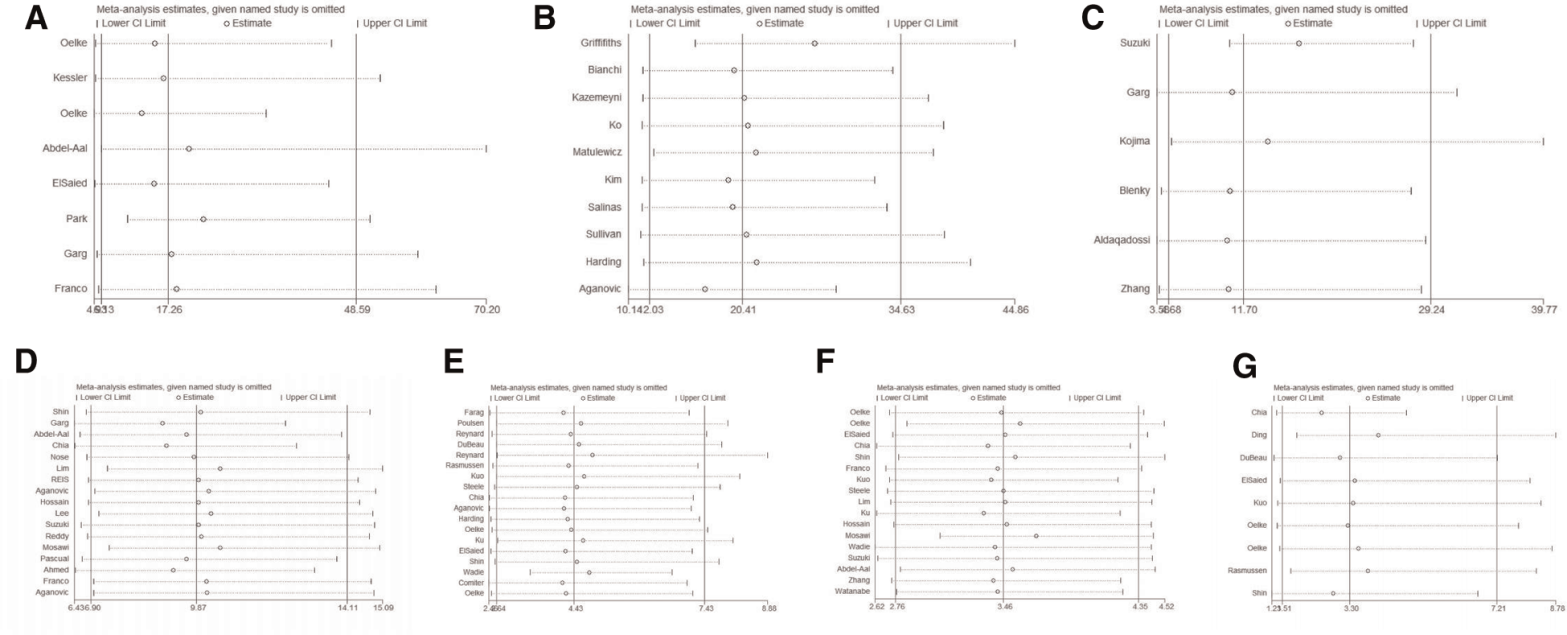
The sensitivity analysis with a 95% confidence interval for DWT (**A**), PCT (**B**), RI (**C**), IPP (**D**), Qmax (**E**), PV (**F**) and PVR (**G**) in the diagnosis of BOO in men.

We conducted sensitivity analysis to evaluate the effect of each individual study on the pooled DOR by removing the eligible study one by one for each of the index tests. The pooled data was not changed substantially by removing any of the eligible studies for these index tests (Figure 6).

### Meta-regression

**Figure 7:**
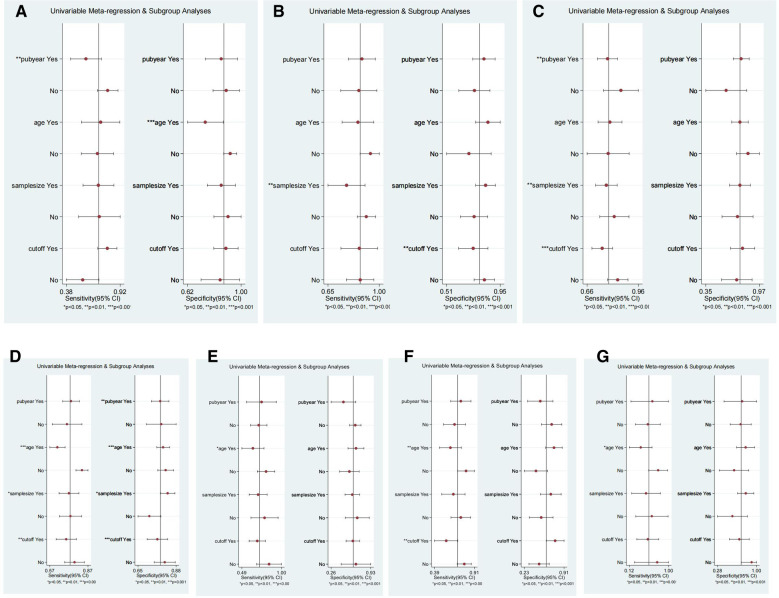
The meta-regression for DWT (**A**), PCT (**B**), RI (**C**), IPP (**D**), Qmax (**E**), PV (**F**) and PVR (**G**) in the diagnosis of BOO in men. Meta-regression was performed according to whether the publication year was after 2010, the mean age was over 65, the sample size was over 100 and to use the most commonly used cut-off.

Next, we performed meta-regression analysis to further identify the sources of heterogeneity, including publication year (pre-2010 vs. post-2010), mean age (≤65 vs. >65), sample size (≤100 vs. >100) and cut-off (most common vs. not). The results of meta-regression analysis are presented in Figure 7. Analysis indicated that some of these four factors may represent the source of heterogeneity for these index tests and that the origin of heterogeneity would be different for different tests.

### Publication bias

**Figure 8:**
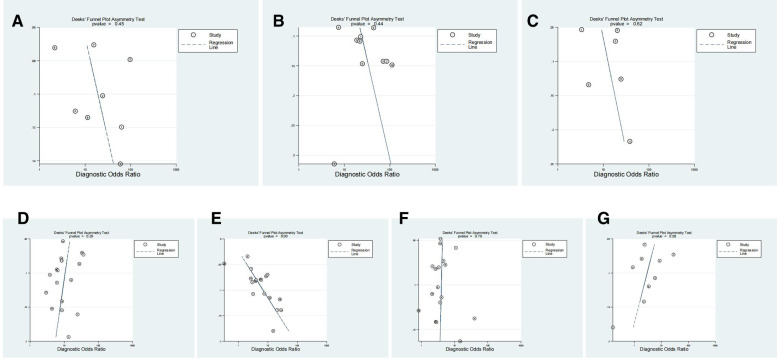
The Deek’s funnel plot for the assessment of publication bias for DWT (**A**), PCT (**B**), RI (**C**), IPP (**D**), Qmax (**E**), PV (**F**) and PVR (**G**) in the diagnosis of BOO in men.

Finally, publication bias was evaluated with a Deeks’ funnel plot and an associated regression test for asymmetry. Funnel plots showed that studies relating to DWT, PCT, RI, IPP, PV and PVR were all evenly distributed on both sides of the regression line (*P* > 0.05; Figure 8). A significant publication bias was identified for studies involving Qmax (*P* < 0.05).

## Discussion

There are several limitations to the use of UDS to diagnose BOO in men, including the invasive nature of this technique, high costs, and side effects. Consequently, there is significant interest in the development of non-invasive methods to diagnose BOO. Over recent years, several different methods have been developed. However, no meta-analysis has been performed to evaluate the diagnostic accuracy of these new non-invasive tests for diagnosing BOO in men. Therefore, we conducted the first meta-analysis to investigate the diagnostic accuracy of seven non-invasive tests by pooling the data and performing quantitative analysis.

Previous studies have investigated the accuracy of non-invasive tools for the diagnosis of BOO in men; however, conclusions remain inconsistent. A previous systematic review reported that PCT, DWT and NIRS had the highest median SSYs ranging from 82% to 85.7% (5). The highest median NPVs ranged from 84% to 89% when using the most common cut-offs. The findings of our present meta-analysis are consistent with these earlier findings in that systematic review-DWT and PCT were the two most promising non-invasive tests.

Our results indicated that the pooled DORs for DWT and PCT were 17.15 (95% CI: 7.09–41.46) and 23.54 (95% CI: 13.56–40.85) respectively. Furthermore, DWT (using 2 mm as the cut-off) exhibited the highest DOR value (32.58, 95% CI: 12.04–88.17). The DOR is one of the most primary indicators for test accuracy and combines data from SSY and SPY into a single number (73). These data suggested that DWT and PCT had the highest accuracies for the diagnosis of BOO in men when compared among the seven non-invasive methods, especially with DWT using the most commonly used cut-off. Furthermore, the SPY (0.89, 95% CI: 0.75–0.96) and AUC (0.90, 95% CI: 0.87–0.92) values for DWT were also the highest. Unlike a conventional ROC curve, which observes the effect of varying cut-offs on SSY and SPY in a single study, each data point represents an individual study in a SROC curve (18). Our results showed that DWT and PCT had a relatively better diagnostic power, with AUCs of 0.87 (95% CI: 0.84–0.90) and 0.88 (95% CI: 0.85–0.91) respectively.

A vital aspect of meta-analysis is to identify the sources of heterogeneity (74). In the present study, heterogeneity tests showed that PCT, Qmax, and PV, had a significant threshold effect. The heterogeneity of the pooled data caused by non-threshold effects was generally high. We did not find that any single study had a significant influence on the pooled data for each type of non-invasive method. However, in our meta-regression analysis, we found that publication year had a significant influence on the SSY of DWT, RI and the SSY of IPP. The mean age exerted an effect on the SSY of IPP, Qmax, PV and PVR. Sample size had an effect on the SSY of PCT, RI, IPP, and the SPY of IPP. The cut-off exerted impact on the SSY of RI, IPP, PV and the SPY of PCT and IPP, thus indicating that researchers need to unify the publication year, mean age, sample size, and/or cut-offs for the corresponding tests affected by these factors in future studies. In addition to this, although the risk of bias was generally low across most domains in most of the studies included in the present analysis, it is worth noting that all of the included studies were non-randomized trials. Only 14 studies applied the blinding methods, the remaining studies were unblinded or unknown. Furthermore, some studies adopted varying thresholds for the same index test and several studies applied different standards to define BOO in men. Therefore, blinding, study design, and the definitions used for BOO may also cause bias in the pooled data.

This was the first meta-analysis to explore the accuracy of non-invasive methods for diagnosing BOO in men. We performed a more comprehensive literature search to provide a newer and more complete dataset so that we could perform quantitative analysis. Nevertheless, there were several limitations. Firstly, we generally observed high levels of heterogeneity in the included studies for each type of index test. Secondly, all of the included studies were non-randomized trials, some were retrospective or were unblinded; this may have induced bias. Thirdly, some of the included studies adopted varying cut-offs or definitions for BOO, this may cause bias. Finally, only studies that were written in English were included; whether studies in other languages could have influenced our results remains unknown.

## Conclusion

This meta-analysis provided relatively good evidence for the diagnostic accuracy of some non-invasive tests, this evidence is not sufficient to provide a new gold standard. However, larger studies, with more stringent methodological standards and larger sample sizes, are now required to better evaluate their value in the diagnosis of BOO in men with LUTS. Of the non-invasive tests tested, DWT and PCT had the highest levels of diagnostic accuracy for diagnosing BOO in men with LUTS. DWT, with a 2 mm cut-off, had the highest level of accuracy. These two methods represent good options as non-invasive tools for evaluating BOO in males.

## Data Availability

The original contributions presented in the study are included in the article/**Supplementary Material**, further inquiries can be directed to the corresponding author/s.
