# Supplementary material for: The diagnostic value of non-invasive methods for diagnosing bladder outlet obstruction in men with lower urinary tract symptoms: A meta-analysis
Source: Front Surg. 2022 Sep 20;9:986679. doi: 10.3389/fsurg.2022.986679 (PMC9632994; doi:10.3389/fsurg.2022.986679)
Supplement: Supplementary file 1 [file Datasheet1.pdf]

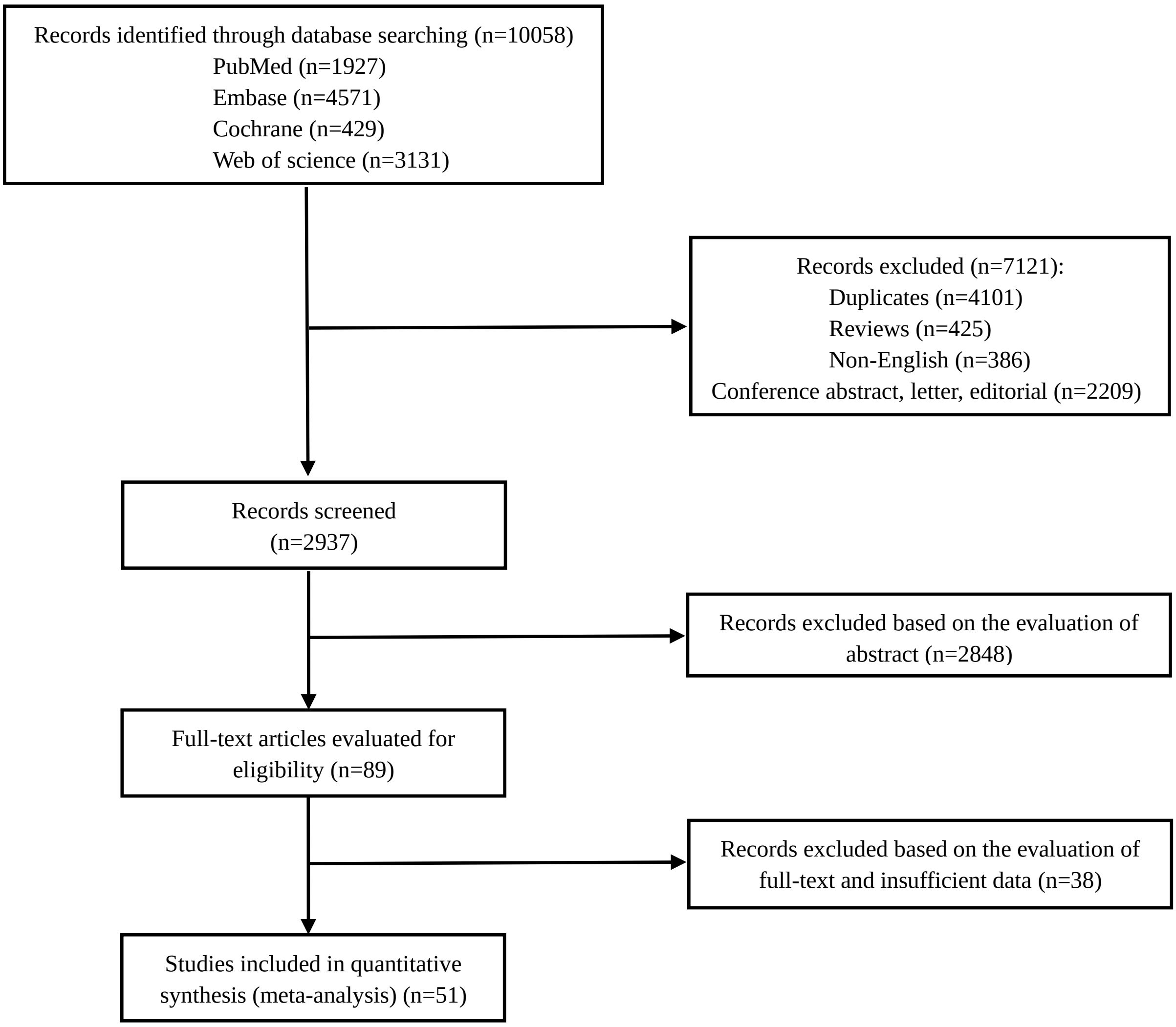
Records identified through database searching (n=10058)
PubMed (n=1927)
Embase (n=4571)
Cochrane (n=429)
Web of science (n=3131)
Records excluded (n=7121):
Duplicates (n=4101)
Reviews (n=425)
Non-English (n=386)
Conference abstract, letter, editorial (n=2209)
Records screened
(n=2937)
Records excluded based on the evaluation of abstract (n=2848)
Full-text articles evaluated for eligibility (n=89)
Records excluded based on the evaluation of full-text and insufficient data (n=38)
Studies included in quantitative synthesis (meta-analysis) (n=51)

	Risk of Bias				Applicability Concerns		
	Patient Selection	Index Test	Reference Standard	Flow and Timing	Patient Selection	Index Test	Reference Standard
Abdel-Aal 2011	+	+	+	+	+	+	+
Aganovic 2004	?	−	−	+	+	+	+
Aganovic 2012 (a)	+	−	−	+	+	+	+
Aganovic 2012 (b)	?	+	−	+	+	+	+
Aganovic 2019	+	+	?	+	+	+	?
Ahmed 2016	?	?	?	+	+	+	+
Aldaqadossi 2012	−	+	+	+	+	+	+
Bianchi 2014	?	+	−	+	+	+	+
Blenky 2003	+	+	+	+	+	+	+
Chia 2003	?	+	+	+	+	+	+
Comiter 1996	?	+	−	+	+	+	−
Ding 1997	?	?	?	+	+	+	+
DuBeau 1998	?	+	−	+	?	+	−
Eisaied 2013	?	+	+	+	+	+	+
Farag 2017	+	?	+	?	+	+	+
Franco 2010	+	+	+	+	+	+	+
Garg 2019	?	+	?	+	+	+	+
Griffiths 2005	?	−	+	−	+	+	+
Harding 2004	?	+	+	+	+	+	+
Hossain 2012	+	+	?	+	+	+	+
Kazemeyni 2015	+	+	+	?	+	+	+
Kessler 2006	+	−	−	+	+	+	+
Kim 2020	?	?	?	+	?	+	+
Ko 2017	?	+	+	+	?	+	+
Kojima 2000	?	+	?	+	+	+	+
Ku 2009	−	+	−	+	+	+	+
Kuo 1999	+	+	−	+	+	+	−
Lee 2016	+	+	?	+	+	+	+
Lim 2006	+	?	?	+	+	+	+
Matulewicz 2015	+	−	+	−	+	+	+
Mosawi 2020	?	+	?	+	+	+	+
Nose 2005	+	+	+	+	+	+	+
Oelke 2002	+	?	?	+	+	+	+
Oelke 2007	+	+	+	+	+	+	+
Park 2020	?	?	?	+	+	+	+
Pascual 2011	+	+	−	+	+	+	+
Poulsen 1994	+	+	−	+	+	+	+
Rasmussen 1999	−	+	?	+	−	+	+
Reddy 2019	+	+	+	+	+	+	+
Reis 2008	+	+	+	+	+	+	+
Reynard 1996	+	+	−	+	+	+	+
Reynard 1998	+	+	?	+	+	+	+
Salinas 2003	+	+	+	+	+	+	+
Shin 2013	+	?	?	+	+	+	+
Steele 2000	?	+	?	+	+	+	+
Sullivan 2000	?	−	?	+	−	+	+
Suzuki 2016	?	+	?	+	+	+	+
Wadie 2021	?	+	?	+	+	+	+
Watanabe 2002	+	?	?	+	+	+	+
Zhang 2012	?	+	+	+	+	+	+
Zhang 2014	+	+	+	+	+	+	+

− High ? Unclear + Low

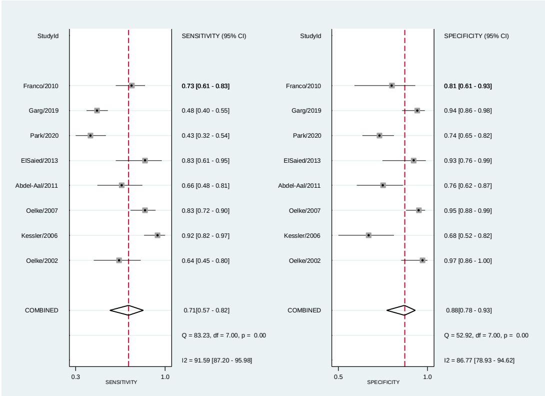
StudyId
SENSITIVITY (95% CI)
Franco/2010 0.73 [0.61 - 0.83]
Garg/2019 0.48 [0.40 - 0.55]
Park/2020 0.43 [0.32 - 0.54]
ElSaied/2013 0.83 [0.61 - 0.95]
Abdel-Aal/2011 0.66 [0.48 - 0.81]
Oelke/2007 0.83 [0.72 - 0.90]
Kessler/2006 0.92 [0.82 - 0.97]
Oelke/2002 0.64 [0.45 - 0.80]
COMBINED 0.71[0.57 - 0.82]
Q = 83.23, df = 7.00, p = 0.00
I2 = 91.59 [87.20 - 95.98]
0.3
1.0
SENSITIVITY
StudyId
SPECIFICITY (95% CI)
Franco/2010 0.81 [0.61 - 0.93]
Garg/2019 0.94 [0.86 - 0.98]
Park/2020 0.74 [0.65 - 0.82]
ElSaied/2013 0.93 [0.76 - 0.99]
Abdel-Aal/2011 0.76 [0.62 - 0.87]
Oelke/2007 0.95 [0.88 - 0.99]
Kessler/2006 0.68 [0.52 - 0.82]
Oelke/2002 0.97 [0.86 - 1.00]
COMBINED 0.88[0.78 - 0.93]
Q = 52.92, df = 7.00, p = 0.00
I2 = 86.77 [78.93 - 94.62]
0.5
1.0
SPECIFICITY

a

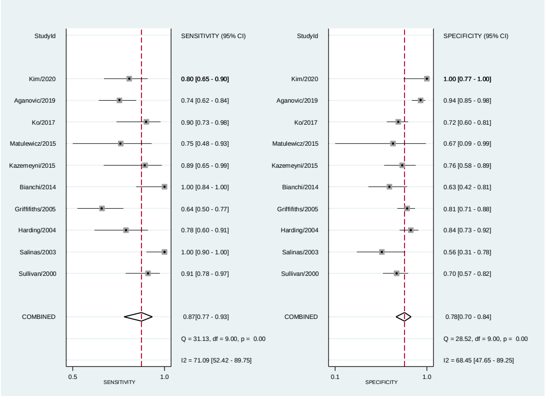
StudyId
SENSITIVITY (95% CI)
Kim/2020 0.80 [0.65 - 0.90]
Aganovic/2019 0.74 [0.62 - 0.84]
Ko/2017 0.90 [0.73 - 0.98]
Matulewicz/2015 0.75 [0.48 - 0.93]
Kazemeyni/2015 0.89 [0.65 - 0.99]
Bianchi/2014 1.00 [0.84 - 1.00]
Griffiths/2005 0.64 [0.50 - 0.77]
Harding/2004 0.78 [0.60 - 0.91]
Salinas/2003 1.00 [0.90 - 1.00]
Sullivan/2000 0.91 [0.78 - 0.97]
COMBINED 0.87[0.77 - 0.93]
Q = 31.13, df = 9.00, p = 0.00
I2 = 71.09 [52.42 - 89.75]
0.5
1.0
SENSITIVITY
StudyId
SPECIFICITY (95% CI)
Kim/2020 1.00 [0.77 - 1.00]
Aganovic/2019 0.94 [0.85 - 0.98]
Ko/2017 0.72 [0.60 - 0.81]
Matulewicz/2015 0.67 [0.09 - 0.99]
Kazemeyni/2015 0.76 [0.58 - 0.89]
Bianchi/2014 0.63 [0.42 - 0.81]
Griffiths/2005 0.81 [0.71 - 0.88]
Harding/2004 0.84 [0.73 - 0.92]
Salinas/2003 0.56 [0.31 - 0.78]
Sullivan/2000 0.70 [0.57 - 0.82]
COMBINED 0.78[0.70 - 0.84]
Q = 28.52, df = 9.00, p = 0.00
I2 = 68.45 [47.65 - 89.25]
0.1
1.0
SPECIFICITY

b

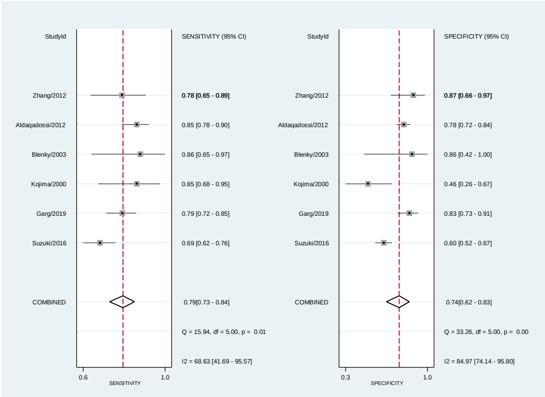
StudyId
SENSITIVITY (95% CI)
Zhang/2012 0.78 [0.65 - 0.89]
Aldaqadossi/2012 0.85 [0.78 - 0.90]
Blenky/2003 0.86 [0.65 - 0.97]
Kojima/2000 0.85 [0.68 - 0.95]
Garg/2019 0.79 [0.72 - 0.85]
Suzuki/2016 0.69 [0.62 - 0.76]
COMBINED 0.79[0.73 - 0.84]
Q = 15.94, df = 5.00, p = 0.01
I2 = 68.63 [41.69 - 95.57]
0.6
1.0
SENSITIVITY
StudyId
SPECIFICITY (95% CI)
Zhang/2012 0.87 [0.66 - 0.97]
Aldaqadossi/2012 0.78 [0.72 - 0.84]
Blenky/2003 0.86 [0.42 - 1.00]
Kojima/2000 0.46 [0.26 - 0.67]
Garg/2019 0.83 [0.73 - 0.91]
Suzuki/2016 0.60 [0.52 - 0.67]
COMBINED 0.74[0.62 - 0.83]
Q = 33.26, df = 5.00, p = 0.00
I2 = 84.97 [74.14 - 95.80]
0.3
1.0
SPECIFICITY

c

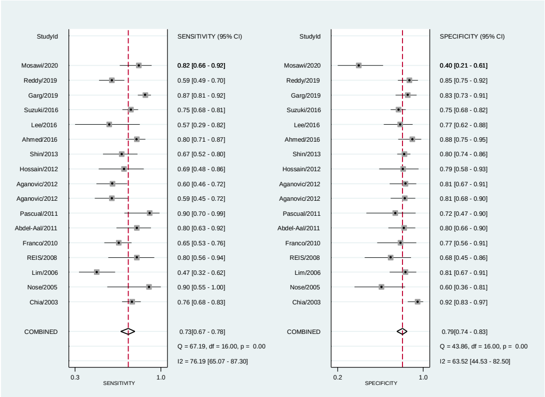
StudyId
SENSITIVITY (95% CI)
Mosawi/2020 0.82 [0.66 - 0.92]
Reddy/2019 0.59 [0.49 - 0.70]
Garg/2019 0.87 [0.81 - 0.92]
Suzuki/2016 0.75 [0.68 - 0.81]
Lee/2016 0.57 [0.29 - 0.82]
Ahmed/2016 0.80 [0.71 - 0.87]
Shin/2013 0.67 [0.52 - 0.80]
Hossain/2012 0.69 [0.48 - 0.86]
Aganovic/2012 0.60 [0.46 - 0.72]
Aganovic/2012 0.59 [0.45 - 0.72]
Pascual/2011 0.90 [0.70 - 0.99]
Abdel-Aal/2011 0.80 [0.63 - 0.92]
Franco/2010 0.65 [0.53 - 0.76]
REIS/2008 0.80 [0.56 - 0.94]
Lim/2006 0.47 [0.32 - 0.62]
Nose/2005 0.90 [0.55 - 1.00]
Chia/2003 0.76 [0.68 - 0.83]
COMBINED 0.73[0.67 - 0.78]
Q = 67.19, df = 16.00, p = 0.00
I2 = 76.19 [65.07 - 87.30]
0.3
1.0
SENSITIVITY
StudyId
SPECIFICITY (95% CI)
Mosawi/2020 0.40 [0.21 - 0.61]
Reddy/2019 0.85 [0.75 - 0.92]
Garg/2019 0.83 [0.73 - 0.91]
Suzuki/2016 0.75 [0.68 - 0.82]
Lee/2016 0.77 [0.62 - 0.88]
Ahmed/2016 0.88 [0.75 - 0.95]
Shin/2013 0.80 [0.74 - 0.86]
Hossain/2012 0.79 [0.58 - 0.93]
Aganovic/2012 0.81 [0.67 - 0.91]
Aganovic/2012 0.81 [0.68 - 0.90]
Pascual/2011 0.72 [0.47 - 0.90]
Abdel-Aal/2011 0.80 [0.66 - 0.90]
Franco/2010 0.77 [0.56 - 0.91]
REIS/2008 0.68 [0.45 - 0.86]
Lim/2006 0.81 [0.67 - 0.91]
Nose/2005 0.60 [0.36 - 0.81]
Chia/2003 0.92 [0.83 - 0.97]
COMBINED 0.79[0.74 - 0.83]
Q = 43.86, df = 16.00, p = 0.00
I2 = 63.52 [44.53 - 82.50]
0.2
1.0
SPECIFICITY

d

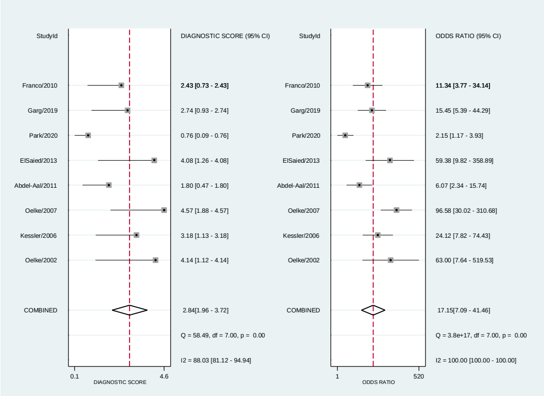
StudyId
DIAGNOSTIC SCORE (95% CI)
Franco/2010
2.43 [0.73 - 2.43]
Garg/2019
2.74 [0.93 - 2.74]
Park/2020
0.76 [0.09 - 0.76]
ElSaied/2013
4.08 [1.26 - 4.08]
Abdel-Aal/2011
1.80 [0.47 - 1.80]
Oelke/2007
4.57 [1.88 - 4.57]
Kessler/2006
3.18 [1.13 - 3.18]
Oelke/2002
4.14 [1.12 - 4.14]
COMBINED
2.84[1.96 - 3.72]
Q = 58.49, df = 7.00, p = 0.00
I2 = 88.03 [81.12 - 94.94]
0.1
4.6
DIAGNOSTIC SCORE
StudyId
ODDS RATIO (95% CI)
Franco/2010
11.34 [3.77 - 34.14]
Garg/2019
15.45 [5.39 - 44.29]
Park/2020
2.15 [1.17 - 3.93]
ElSaied/2013
59.38 [9.82 - 358.89]
Abdel-Aal/2011
6.07 [2.34 - 15.74]
Oelke/2007
96.58 [30.02 - 310.68]
Kessler/2006
24.12 [7.82 - 74.43]
Oelke/2002
63.00 [7.64 - 519.53]
COMBINED
17.15[7.09 - 41.46]
Q = 3.8e+17, df = 7.00, p = 0.00
I2 = 100.00 [100.00 - 100.00]
1
520
ODDS RATIO

a

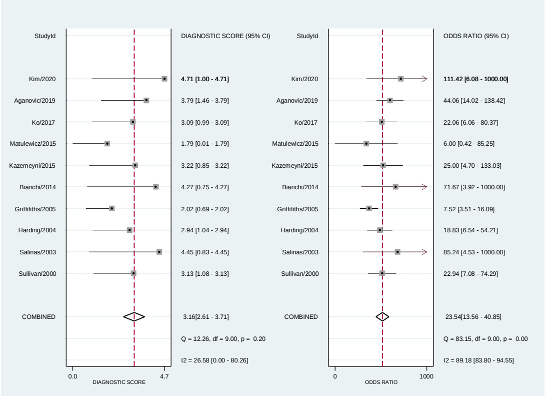
StudyId
DIAGNOSTIC SCORE (95% CI)
Kim/2020
4.71 [1.00 - 4.71]
Aganovic/2019
3.79 [1.46 - 3.79]
Ko/2017
3.09 [0.99 - 3.09]
Matulewicz/2015
1.79 [0.01 - 1.79]
Kazemeyni/2015
3.22 [0.85 - 3.22]
Bianchi/2014
4.27 [0.75 - 4.27]
Griffifiths/2005
2.02 [0.69 - 2.02]
Harding/2004
2.94 [1.04 - 2.94]
Salinas/2003
4.45 [0.83 - 4.45]
Sullivan/2000
3.13 [1.08 - 3.13]
COMBINED
3.16[2.61 - 3.71]
Q = 12.26, df = 9.00, p = 0.20
I2 = 26.58 [0.00 - 80.26]
0.0
4.7
DIAGNOSTIC SCORE
StudyId
ODDS RATIO (95% CI)
Kim/2020
111.42 [6.08 - 1000.00]
Aganovic/2019
44.06 [14.02 - 138.42]
Ko/2017
22.06 [6.06 - 80.37]
Matulewicz/2015
6.00 [0.42 - 85.25]
Kazemeyni/2015
25.00 [4.70 - 133.03]
Bianchi/2014
71.67 [3.92 - 1000.00]
Griffifiths/2005
7.52 [3.51 - 16.09]
Harding/2004
18.83 [6.54 - 54.21]
Salinas/2003
85.24 [4.53 - 1000.00]
Sullivan/2000
22.94 [7.08 - 74.29]
COMBINED
23.54[13.56 - 40.85]
Q = 83.15, df = 9.00, p = 0.00
I2 = 89.18 [83.80 - 94.55]
0
1000
ODDS RATIO

b

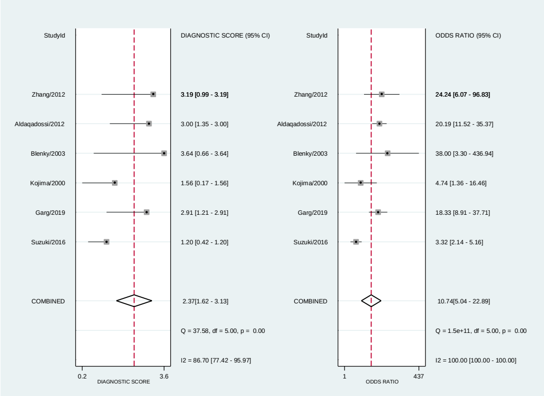
StudyId
DIAGNOSTIC SCORE (95% CI)
Zhang/2012
3.19 [0.99 - 3.19]
Aldaqadossi/2012
3.00 [1.35 - 3.00]
Blenky/2003
3.64 [0.66 - 3.64]
Kojima/2000
1.56 [0.17 - 1.56]
Garg/2019
2.91 [1.21 - 2.91]
Suzuki/2016
1.20 [0.42 - 1.20]
COMBINED
2.37[1.62 - 3.13]
Q = 37.58, df = 5.00, p = 0.00
I2 = 86.70 [77.42 - 95.97]
0.2
3.6
DIAGNOSTIC SCORE
StudyId
ODDS RATIO (95% CI)
Zhang/2012
24.24 [6.07 - 96.83]
Aldaqadossi/2012
20.19 [11.52 - 35.37]
Blenky/2003
38.00 [3.30 - 436.94]
Kojima/2000
4.74 [1.36 - 16.46]
Garg/2019
18.33 [8.91 - 37.71]
Suzuki/2016
3.32 [2.14 - 5.16]
COMBINED
10.74[5.04 - 22.89]
Q = 1.5e+11, df = 5.00, p = 0.00
I2 = 100.00 [100.00 - 100.00]
1
437
ODDS RATIO

c

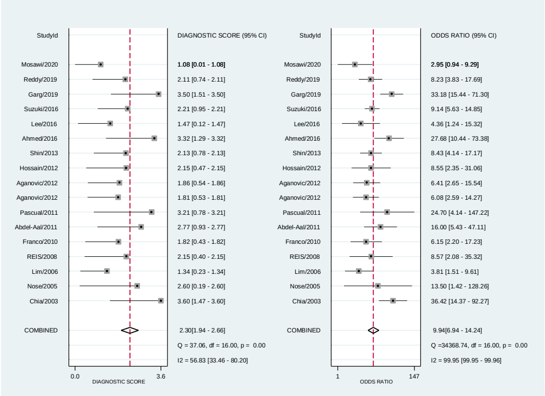
StudyId
DIAGNOSTIC SCORE (95% CI)
Mosawi/2020
1.08 [0.01 - 1.08]
Reddy/2019
2.11 [0.74 - 2.11]
Garg/2019
3.50 [1.51 - 3.50]
Suzuki/2016
2.21 [0.95 - 2.21]
Lee/2016
1.47 [0.12 - 1.47]
Ahmed/2016
3.32 [1.29 - 3.32]
Shin/2013
2.13 [0.78 - 2.13]
Hossain/2012
2.15 [0.47 - 2.15]
Aganovic/2012
1.86 [0.54 - 1.86]
Aganovic/2012
1.81 [0.53 - 1.81]
Pascual/2011
3.21 [0.78 - 3.21]
Abdel-Aal/2011
2.77 [0.93 - 2.77]
Franco/2010
1.82 [0.43 - 1.82]
REIS/2008
2.15 [0.40 - 2.15]
Lim/2006
1.34 [0.23 - 1.34]
Nose/2005
2.60 [0.19 - 2.60]
Chia/2003
3.60 [1.47 - 3.60]
COMBINED
2.30[1.94 - 2.66]
Q = 37.06, df = 16.00, p = 0.00
I2 = 56.83 [33.46 - 80.20]
0.0
3.6
DIAGNOSTIC SCORE
StudyId
ODDS RATIO (95% CI)
Mosawi/2020
2.95 [0.94 - 9.29]
Reddy/2019
8.23 [3.83 - 17.69]
Garg/2019
33.18 [15.44 - 71.30]
Suzuki/2016
9.14 [5.63 - 14.85]
Lee/2016
4.36 [1.24 - 15.32]
Ahmed/2016
27.68 [10.44 - 73.38]
Shin/2013
8.43 [4.14 - 17.17]
Hossain/2012
8.55 [2.35 - 31.06]
Aganovic/2012
6.41 [2.65 - 15.54]
Aganovic/2012
6.08 [2.59 - 14.27]
Pascual/2011
24.70 [4.14 - 147.22]
Abdel-Aal/2011
16.00 [5.43 - 47.11]
Franco/2010
6.15 [2.20 - 17.23]
REIS/2008
8.57 [2.08 - 35.32]
Lim/2006
3.81 [1.51 - 9.61]
Nose/2005
13.50 [1.42 - 128.26]
Chia/2003
36.42 [14.37 - 92.27]
COMBINED
9.94[6.94 - 14.24]
Q =34368.74, df = 16.00, p = 0.00
I2 = 99.95 [99.95 - 99.96]
1
147
ODDS RATIO

d

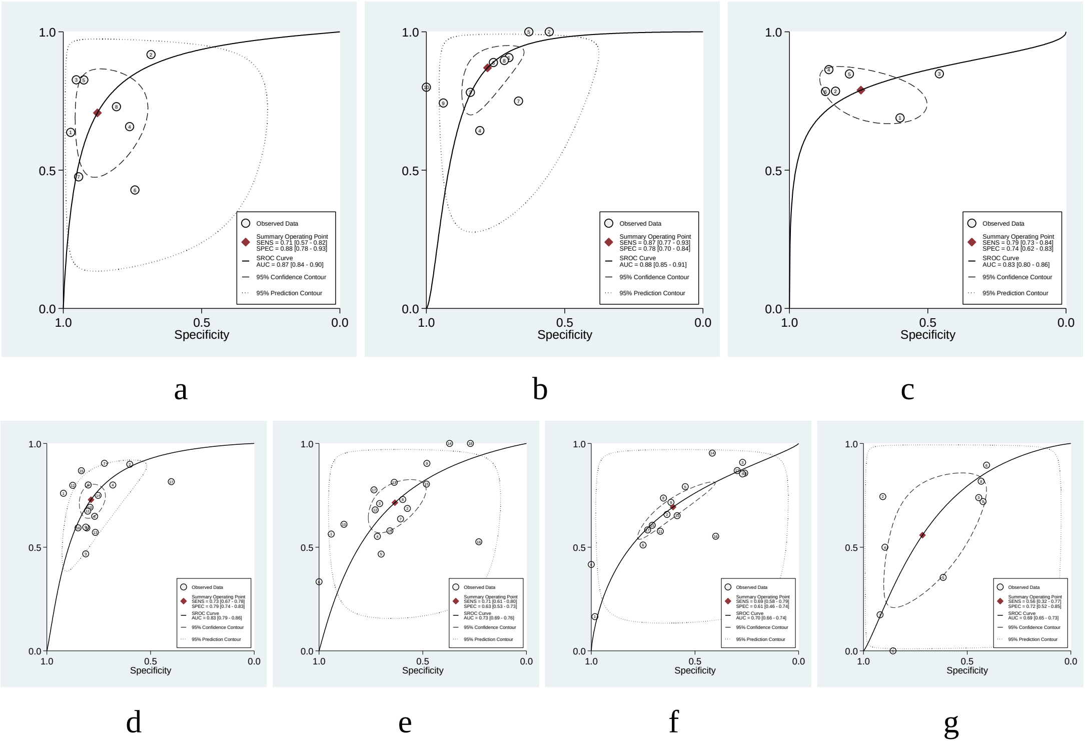

a
Observed Data
Summary Operating Point
SENS = 0.71 [0.57 - 0.82]
SPEC = 0.88 [0.78 - 0.93]
SROC Curve
AUC = 0.87 [0.84 - 0.90]
95% Confidence Contour
95% Prediction Contour
Specificity
b
Observed Data
Summary Operating Point
SENS = 0.87 [0.77 - 0.93]
SPEC = 0.78 [0.70 - 0.84]
SROC Curve
AUC = 0.88 [0.85 - 0.91]
95% Confidence Contour
95% Prediction Contour
Specificity
c
Observed Data
Summary Operating Point
SENS = 0.79 [0.73 - 0.84]
SPEC = 0.74 [0.62 - 0.83]
SROC Curve
AUC = 0.83 [0.80 - 0.86]
95% Confidence Contour
95% Prediction Contour
Specificity
d
Observed Data
Summary Operating Point
SENS = 0.73 [0.67 - 0.78]
SPEC = 0.79 [0.74 - 0.83]
SROC Curve
AUC = 0.83 [0.79 - 0.86]
95% Confidence Contour
95% Prediction Contour
Specificity
e
Observed Data
Summary Operating Point
SENS = 0.71 [0.61 - 0.80]
SPEC = 0.63 [0.53 - 0.73]
SROC Curve
AUC = 0.73 [0.69 - 0.76]
95% Confidence Contour
95% Prediction Contour
Specificity
f
Observed Data
Summary Operating Point
SENS = 0.69 [0.58 - 0.79]
SPEC = 0.61 [0.46 - 0.74]
SROC Curve
AUC = 0.70 [0.66 - 0.74]
95% Confidence Contour
95% Prediction Contour
Specificity
g
Observed Data
Summary Operating Point
SENS = 0.56 [0.32 - 0.77]
SPEC = 0.72 [0.52 - 0.85]
SROC Curve
AUC = 0.69 [0.65 - 0.73]
95% Confidence Contour
95% Prediction Contour
Specificity

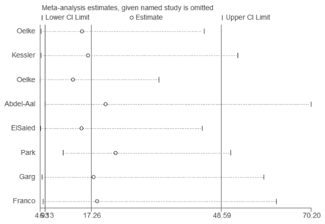

Meta-analysis estimates, given named study is omitted
Lower CI Limit
Estimate
Upper CI Limit
Oelke
Kessler
Oelke
Abdel-Aal
ElSaied
Park
Garg
Franco
17.26
48.59
70.20

a

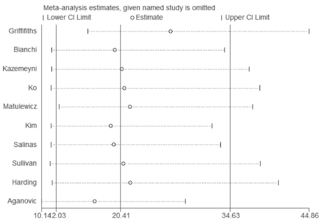

Meta-analysis estimates, given named study is omitted
Lower CI Limit
Estimate
Upper CI Limit
Griffifiths
Bianchi
Kazemeyni
Ko
Matulewicz
Kim
Salinas
Sullivan
Harding
Aganovic
20.41
34.63
44.86

b

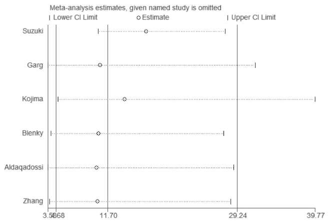

Meta-analysis estimates, given named study is omitted
Lower CI Limit
Estimate
Upper CI Limit
Suzuki
Garg
Kojima
Blenky
Aldaqadossi
Zhang
11.70
29.24
39.77

c

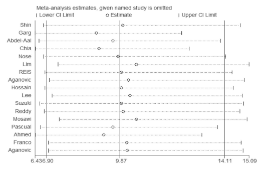

d

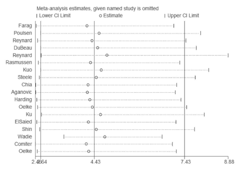

e

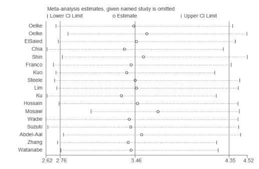

f

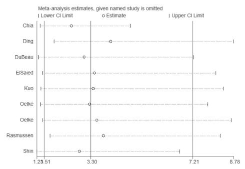

g

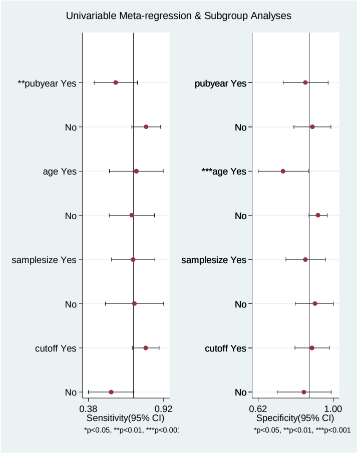
Univariable Meta-regression & Subgroup Analyses
**pubyear Yes
No
age Yes
No
samplesize Yes
No
cutoff Yes
No
0.38
0.92
Sensitivity(95% CI)
pubyear Yes
No
***age Yes
No
samplesize Yes
No
cutoff Yes
No
0.62
1.00
Specificity(95% CI)
*p<0.05, **p<0.01, ***p<0.001

a

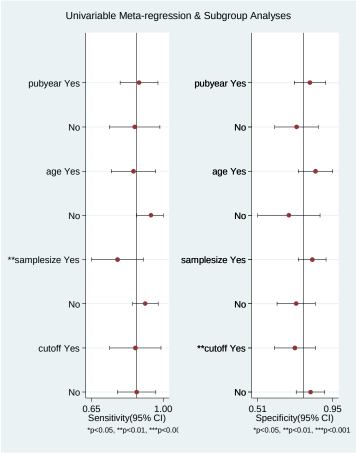
Univariable Meta-regression & Subgroup Analyses
pubyear Yes
No
age Yes
No
**samplesize Yes
No
cutoff Yes
No
0.65
1.00
Sensitivity(95% CI)
pubyear Yes
No
age Yes
No
samplesize Yes
No
**cutoff Yes
No
0.51
0.95
Specificity(95% CI)
*p<0.05, **p<0.01, ***p<0.001

b

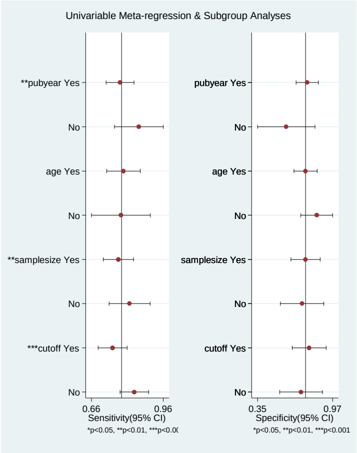
Univariable Meta-regression & Subgroup Analyses
**pubyear Yes
No
age Yes
No
**samplesize Yes
No
***cutoff Yes
No
0.66
0.96
Sensitivity(95% CI)
pubyear Yes
No
age Yes
No
samplesize Yes
No
cutoff Yes
No
0.35
0.97
Specificity(95% CI)
*p<0.05, **p<0.01, ***p<0.001

c

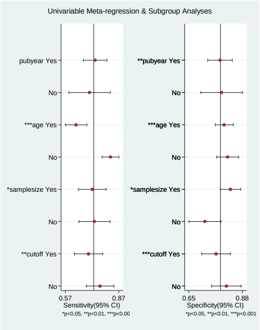
Univariable Meta-regression & Subgroup Analyses
pubyear Yes
No
***age Yes
No
*samplesize Yes
No
**cutoff Yes
No
0.57
0.87
Sensitivity(95% CI)
**pubyear Yes
No
***age Yes
No
*samplesize Yes
No
***cutoff Yes
No
0.65
0.88
Specificity(95% CI)

d

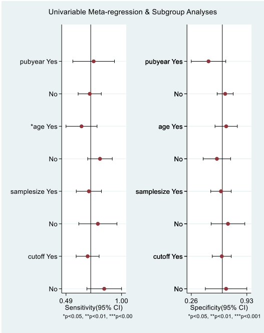
Univariable Meta-regression & Subgroup Analyses
pubyear Yes
No
*age Yes
No
samplesize Yes
No
cutoff Yes
No
0.49
1.00
Sensitivity(95% CI)
pubyear Yes
No
age Yes
No
samplesize Yes
No
cutoff Yes
No
0.26
0.93
Specificity(95% CI)

e

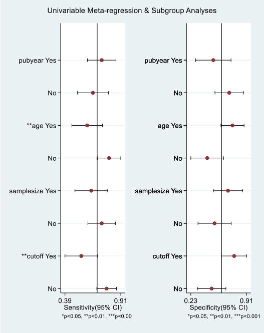
Univariable Meta-regression & Subgroup Analyses
pubyear Yes
No
**age Yes
No
samplesize Yes
No
**cutoff Yes
No
0.39
0.91
Sensitivity(95% CI)
pubyear Yes
No
age Yes
No
samplesize Yes
No
cutoff Yes
No
0.23
0.91
Specificity(95% CI)

f

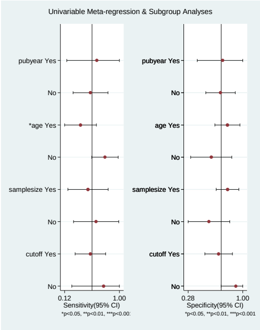
Univariable Meta-regression & Subgroup Analyses
pubyear Yes
No
*age Yes
No
samplesize Yes
No
cutoff Yes
No
0.12
1.00
Sensitivity(95% CI)
pubyear Yes
No
age Yes
No
samplesize Yes
No
cutoff Yes
No
0.28
1.00
Specificity(95% CI)

g

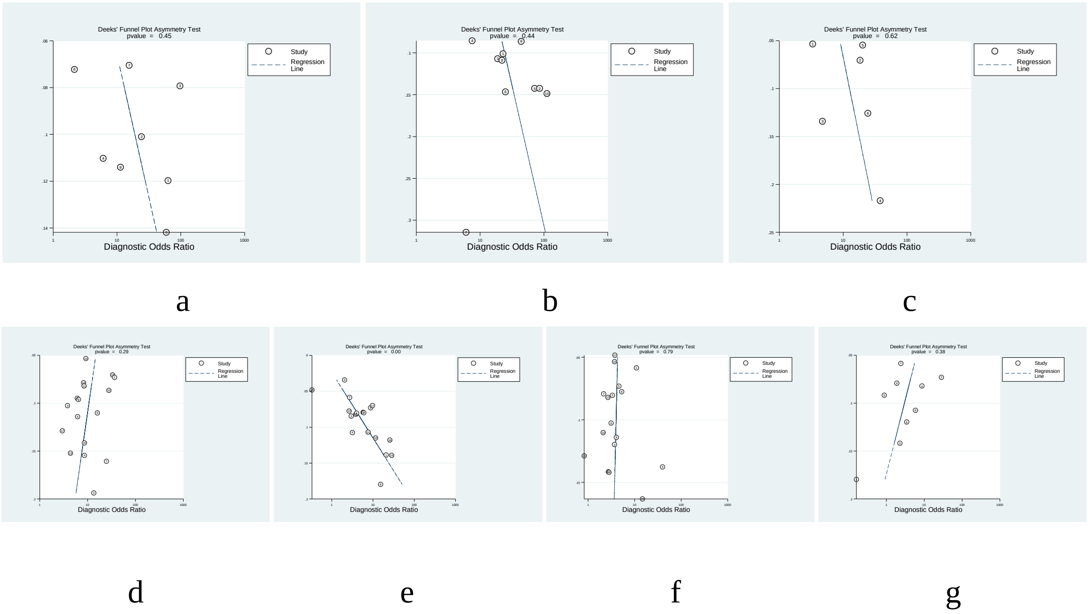

Deeks' Funnel Plot Asymmetry Test
pvalue = 0.45
Study
Regression Line
Diagnostic Odds Ratio
Deeks' Funnel Plot Asymmetry Test
pvalue = 0.44
Study
Regression Line
Diagnostic Odds Ratio
Deeks' Funnel Plot Asymmetry Test
pvalue = 0.62
Study
Regression Line
Diagnostic Odds Ratio
a
b
c
Deeks' Funnel Plot Asymmetry Test
pvalue = 0.29
Study
Regression Line
Diagnostic Odds Ratio
Deeks' Funnel Plot Asymmetry Test
pvalue = 0.00
Study
Regression Line
Diagnostic Odds Ratio
Deeks' Funnel Plot Asymmetry Test
pvalue = 0.79
Study
Regression Line
Diagnostic Odds Ratio
Deeks' Funnel Plot Asymmetry Test
pvalue = 0.38
Study
Regression Line
Diagnostic Odds Ratio
d
e
f
g
